# Which Environmental Factor Is Correlated with Long-Term Multiple Sclerosis Incidence Trends: Ultraviolet B Radiation or Geomagnetic Disturbances?

**DOI:** 10.1155/2017/4960386

**Published:** 2017-10-24

**Authors:** Seyed Aidin Sajedi, Fahimeh Abdollahi

**Affiliations:** ^1^Neuroscience Research Center, Faculty of Medicine, Golestan University of Medical Sciences, Gorgan, Iran; ^2^Department of Neurology, Sayyad Shirazi Hospital, Golestan University of Medical Sciences, Gorgan, Iran; ^3^Multiple Sclerosis Center, Golestan Hospital, Ahvaz, Iran; ^4^Department of Internal Medicine, Sayyad Shirazi Hospital, Golestan University of Medical Sciences, Gorgan, Iran

## Abstract

**Background:**

Insufficient received ultraviolet B radiation (UV) is regarded as the main environmental risk factor (RF) for MS in vitamin D deficiency hypothesis. Nevertheless, geomagnetic disturbance (GMD) has also been proposed as a potential trigger for MS in GMD hypothesis. The aim of this study was to investigate which of these mentioned RF is correlated with long-term ultradecadal MS incidence.

**Methods:**

After a systematic search, long-term incidence reports of the United Kingdom (UK), Denmark, Tayside County, Nordland County, the Orkney, and Shetland Islands were selected for this retrospective time-series study. Possible lead-lag relationships between MS incidence, GMD, and UV were evaluated by cross-correlation analysis.

**Results:**

Significant positive correlations between GMD and MS incidence were seen in Tayside County (at lag of 2 years: *r*_*S*_ = 0.38), Denmark (peak correlation at lag of 2 years: *r*_*S*_ = 0.53), and UK (at lag of 1 year: *r*_*S*_ = 0.50). We found a positive correlation between received UV and MS incidences in the Nordland at lag of 1 year (*r*_*S*_ = 0.49).

**Conclusion:**

This study found significant positive correlations between alterations in GMD with alterations in long-term MS incidence in three out of six studied locations and supports the GMD hypothesis. The observed significant correlation between MS and UV is positive; hence it is not supportive for UV related vitamin D deficiency hypothesis.

## 1. Introduction

Multiple sclerosis (MS) is the most common disabling neurological disorder in the young adults. Genetic studies of twins have revealed that the role of genes in MS is utmost 30% as a factor of susceptibility, and considering the special epidemiological features of MS, the remained roles are played by one or multiple nongenetic, environmental triggers. The actual nature of the environmental risk factor(s) of MS is still subject of debate. In recent years, the majority of MS researchers have focused on vitamin D deficiency as a potential risk factor for this disease. Basically, the vitamin D deficiency hypothesis (VDH) results from the epidemiological features of MS, especially its famous latitudinal gradient of prevalence and the effect of month of the birth. While we know that the climate and temperature are not the risk factors for MS, it seems logical that biomedical researchers suppose that the only remained environmental factor that significantly changes with the latitude and seasons, with the ability to cause a known biological effect, is the received solar ultraviolet radiation (UV). Nevertheless, it is necessary to be considered that there is another solar-terrestrial related phenomenon that may describe MS epidemiological features and has not been well studied in this regard.

The geomagnetic field is a fundamental nature of the planet that is produced by the geodynamo of the Earth's outer core. All living beings live from birth to death in this field, and many species sense it and use it for navigation and immigration. There is evidence that changes in this field can affect biological systems [1]. The presence of this field is vital for saving the atmosphere and the life on our planet from the dangerous particles of solar winds and cosmic radiations. While geomagnetic field deflects the solar wind particles, any changes in the density or the velocity of the solar wind interact with the magnetosphere and cause temporary alterations in the field that is measurable at the Earth surface [2]. This phenomenon is called geomagnetic disturbance (GMD) or geomagnetic activity.

There is a growing body of evidence that GMD affects the human health. The associations between GMD and increased risk of myocardial infarction, suicide, and stroke have been reported, previously [3–7]. In spite of primary suggestions by some researchers in the past decades [8, 9], we found that this issue as a possible environmental risk factor for MS has been neglected, and no comprehensive hypothesis has been designed to explain the potential relationship between GMD and MS.

Studying the physics of GMD and the epidemiology of MS, concomitantly, we noticed potential abilities of a GMD based hypothesis to describe epidemiological features of MS. Accordingly, we framed a comprehensive GMD hypothesis to describe MS epidemiological features. The basic core of the GMD hypothesis is that “vulnerable individuals based on their genetical susceptibility of cell response to magnetic field alterations would suffer from MS attacks in the geographical locations and time periods that GMD matches the sensitivity of their adaptive cell immunity and/or their blood-brain barrier, provided it lasts long enough to stimulate various elements of these systems to enhance immune cell entering to CNS and activating without the presence of costimulatory signals” [10, 11].

The GMD hypothesis has been tested by an extensive ecological study to find whether it can describe MS prevalence distribution [10]. The result pointed out that the GMD hypothesis can explain MS prevalence distribution much better than the VDH. We found that the angular distance to geomagnetic 60-degree latitude (GML60) can describe the MS prevalence significantly better than the angular distance to the equator (i.e., geographical latitude) that is the base element of the VDH. We also have discussed in detail how the GMD hypothesis can also provide the explanation about the effect of immigration, the cause of gradual attenuation of MS prevalence gradient, the effect of time of birth, and the historical changes in MS incidence and epidemics [10, 18].

Moreover, we tested the probable association of GMDs and long-term MS incidence by a retrospective time-series study on recent reports of MS incidence in Tehran and western Greece during the 23rd solar cycle (1996–2008). We found significant correlations between GMDs and alterations of MS incidence in these two locations [19]. Nevertheless that time-series study supported the ability of the GMD hypothesis in describing MS incidence alterations, its main limitation was the fact that Iran and Greece do not have perfect long-term MS registries, and, more importantly, it has not been investigated whether VDH can also explain their MS alterations or not. Therefore, we planned to do a similar study on reported long-term MS incidence of high-latitude areas, especially from the countries with better MS registries and closer to GML60. Moreover, we tried to provide essential UV data to compare the GMD hypothesis and the VDH in this regard, as possible.

## 2. Method and Materials

Our aim was to conduct a retrospective time-series analysis on the long-term MS incidence data of high-latitude European countries (near to GML60) to test the existence of a possible association between MS incidence with GMDs and alterations in the received local UV. For conducting this study, we needed three sets of data including long-term MS incidence data, long-term GMD data, and long-term local received UV data.

### 2.1. Long-Term MS Incidence Data

We searched published works up to 2015 in the PubMed with keywords “Multiple sclerosis” and “Incidence” as follows:

  “(((Multiple sclerosis [Title]) AND incidence [Title])) AND* country name*”

Instead of the* country name*, the names of countries near GML60 were put, including Finland, Norway, Sweden, Denmark, Netherland, and the United Kingdom and its northern parts involving Scotland and Northern Ireland. The search result was evaluated to find studies that had reported annual incidence (new cases) or incidence rate of MS for at least 20 consecutive years. Studies with low-resolution data that just reported period incidence were omitted from the list. In addition to the PubMed search, the authors' archive of MS epidemiological researches was also used for this aim.

**Figure 1:**
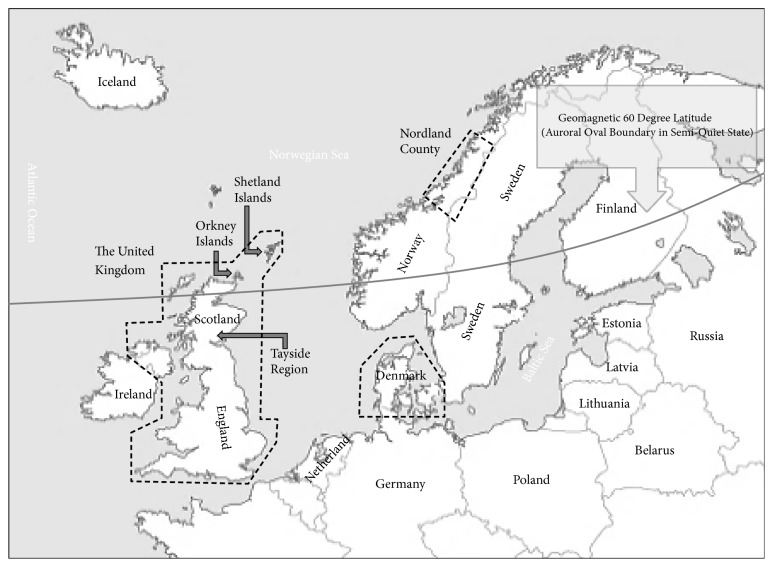
Geographical location of the studied regions and their distance from GML60.

**Figure 2:**
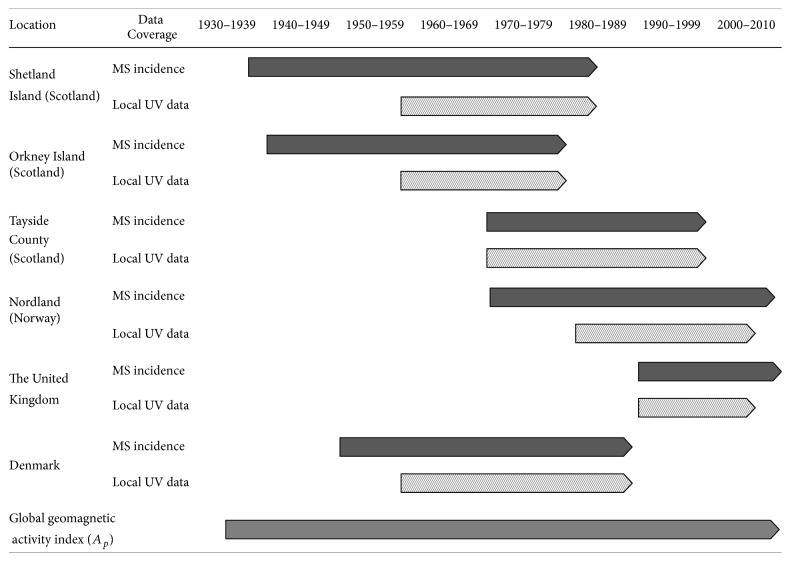
Data coverage of MS incidence, GMD, and received local UV.

Based on this search strategy, six published reports including long-term incidence report of the United Kingdom [17], Denmark [16], Tayside County (Scotland) [14], Nordland County (Norway) [15], the Orkney Islands [12], and the Shetland Islands (Scotland) [13] were selected for this study.  Figure 1 shows the geographical location of the studied regions and their distance from GML60.  Figure 2 illustrates the coverage of the data of those reports.

Except for the report of Nordland, other studies had illustrated the incidence data in the form of figures. Therefore, we extracted and calculated the total (both sexes) MS incidence from their figures. In the reports of Nordland and the Orkney Islands, researchers had described the annual incidence as the number of new cases, but incidence rates were reported just for periods. In these cases, by using their reported population of the regions for each period and annual new cases within that period, we estimated the annual incidence rates and used these estimates in the analyses.

### 2.2. GMD Data

Thirteen observatories in both hemispheres record every 3 hours the variations of the horizontal component of the geomagnetic field. These variations are recorded based on a logarithmic scale that is called *K* indices. The arithmetic mean of the *K* values scaled at the 13 observatories gives Kp. The linear equivalent of Kp is *A* index. Ap index is a daily averaged planetary *A* index of GMD based on data from a set of specific stations and is frequently used as the foremost GMD index in space-weather studies. We used Ap index as the main GMD index in this study. Ap data were extracted from Goddard Space Flight Center's OMNI data set through OMNIWeb [20] and from geomagnetic indices database of National Geophysical Data Center [21].

### 2.3. UV Data

We used two sources for obtaining long-term local UV data: the PROMOTE UV record and the COST 726 project data. PROMOTE is a long-term multisensorial UV record [22]. This project includes calculated ground received UV data of about 100 sites from 1983 to 2007. The advantage of PROMOTE project is the fact that it provides the data of received action spectrum of previtamin D_3_ in human skin (i.e., vitamin D weighted daily dose).

The COST Action 726 project is a database that involves long-term changes and climatology of UV radiation over Europe [23]. It provides data of the received UV based on the action spectrum for ultraviolet induced erythema in human skin (i.e., erythemally weighted daily UV doses). The advantage of the COST 726 is the fact that it is not site-specific and returns the data of the region of interest based on its latitude and longitude with a 1°  × 1° resolution. It has a longer coverage than PROMOTE and includes daily received UV data from 1958 to 2001. Nevertheless, its data for locations beyond 61° latitude includes significant data loss. Accordingly, in the case of Nordland (located at 67° latitude), due to the significant data loss of the COST 726, we used the nearest PROMOTE site (Alomar, located at 69° latitude) as the nearest estimate of the local long-term received UV data.

Because of the fact that the reports of the UK and Denmark include MS incidence data of a large area (the whole country), we used multiple sites of the UV records throughout these countries and calculated their averaged received UV in the period of interests as an estimate of the whole country received UV.

### 2.4. Statistical Analysis

Possible lead-lag relationships among mentioned variables were evaluated by means of cross-correlation analysis [24, 25] for lags between zero and five years. Accordingly, we conducted correlational analysis of [*X*(*t*), *Y*(*t* − *T*)] where *X* is the MS incidence in the region of interest, *Y* is the yearly average of Ap or UV, *t* is the time (year), and *T* is the lag (0–5 years).

By default, cross-correlation function (CCF) calculates Pearson's correlation coefficient (*r*) for determined lags; however, features of the interested data necessitated analyzing with nonparametric method. Therefore, we ranked all variables from the smallest to the largest and then conducted cross-correlation analyses on the ranked variables data to achieve a nonparametric cross-correlation coefficient (*r*_*s*_) result. Nevertheless the analyses were done on the ranked data; as the original data seems nonstationary, detrending was exerted on the ranked data during the cross-correlation analysis by natural log transformation. Statistical analysis was performed by using SPSS 16.0 (SPSS Inc., Chicago, IL, USA). We considered the cross-correlation result significant when the value of CCF was outside the estimated 95% confidence limits of CCF for that lagged time.

## 3. Result

### 3.1. The Orkney Islands

#### 3.1.1. GMD

**Figure 3:**
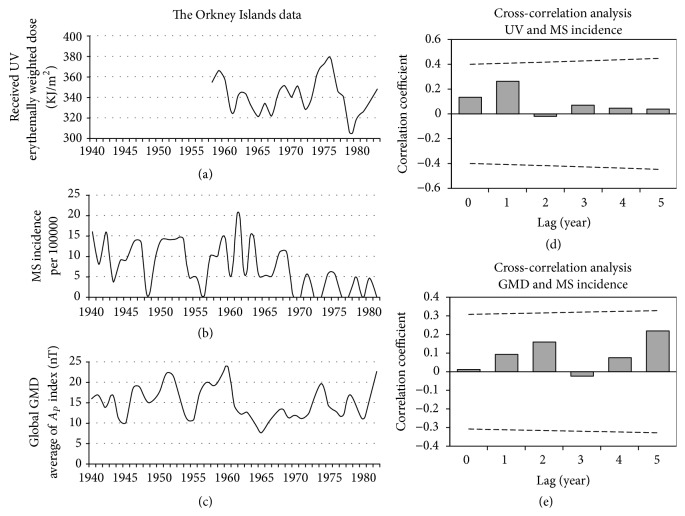
The Orkney Islands data. Panel (a): annual average of the received UV (1958–82). Panel (b): MS incidence of the Orkney Islands (1941–82), adapted from the report of Cook et al. [12]. Panel (c): annual average of global GMD based on the *A*_*p*_ index (1941–82). Panel (d): the result of lagged correlation between received UV and MS incidence. Panel (e): the result of lagged correlation between GMD and MS incidence. The dashed lines in those panels (d and e) are the threshold for significant correlation. The horizontal solid lines in these panels indicate coefficient of correlation (*r*) = 0.

Figure 3 plots the correlation coefficients (*r*_*S*_) between Ap with the reported MS incidence of Orkney (1941–82). We did not find a significant correlation between GMD with MS incidence at lags of 0 to 5 years in this area (Figure 3).

#### 3.1.2. UV

UV data for this region is extracted from COST 726 and covers the years after 1958. We did not find a significant correlation between received UV (1958–82) with MS incidence (1958–82) at lags of 0 to 5 years in this area (Figure 3).

### 3.2. The Shetland Islands

**Figure 4:**
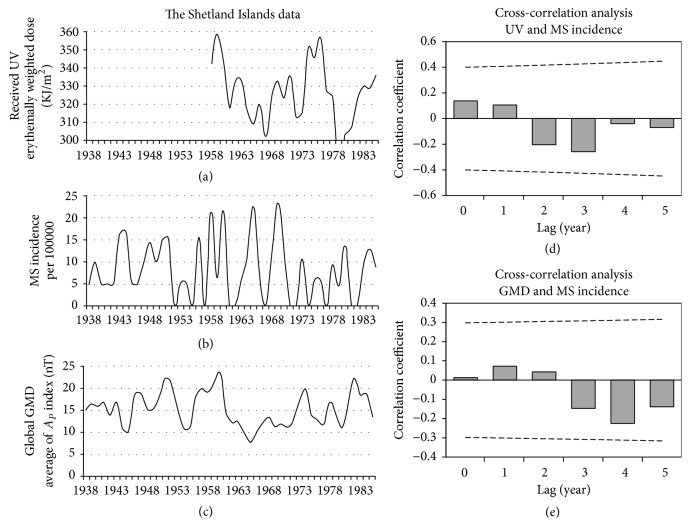
The Shetland Islands data. Panel (a): annual average of the received UV (1958–85). Panel (b): MS incidence of the Shetland Islands (1938–85), adapted from the report of Cook et al. [13]. Panel (c): annual average of global GMD based on the *A*_*p*_ index (1938–85). Panel (d): the result of lagged correlation between received UV and MS incidence. Panel (e): the result of lagged correlation between GMD and MS incidence. The dashed lines in those panels (d and e) are the threshold for significant correlation. The horizontal solid lines in these panels indicate coefficient of correlation (*r*) = 0.

The cross-correlation analysis on Ap with MS incidence (1938–1985) did not show significant correlation at lags of 0 to 5 years. Moreover, we did not find a significant correlation between received UV data (1958–1985) and MS incidence (1958–1985) in this region (Figure 4).

### 3.3. Tayside Region

#### 3.3.1. GMD

**Figure 5:**
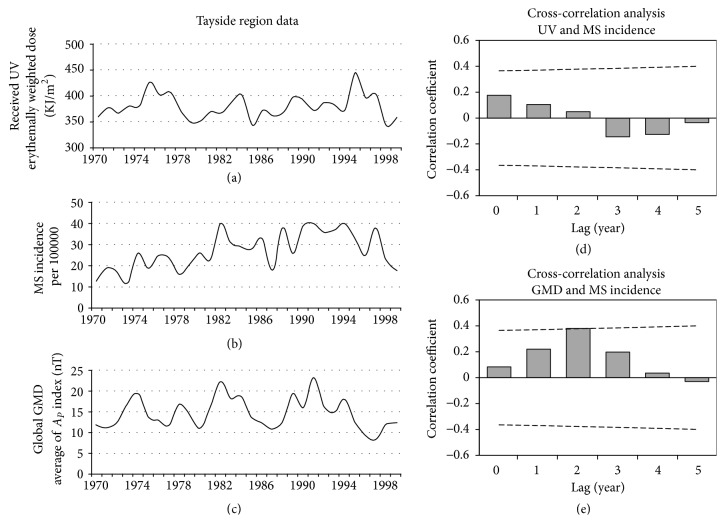
Tayside region data. Panel (a): annual average of the received UV (1970–99). Panel (b): MS incidence of the Tayside region (1970–99), adapted from the report of Donnan et al. [14]. Panel (c): annual average of global GMD based on the *A*_*p*_ index (1970–99). Panel (d): the result of lagged correlation between received UV and MS incidence. Panel (e): the result of lagged correlation between GMD and MS incidence. The dashed lines in those panels (d and e) are the threshold for significant correlation. The horizontal solid lines in these panels indicate coefficient of correlation (*r*) = 0.

The cross-correlation analysis showed significant positive correlation between Ap and Tayside MS incidence (1970–1999) at a lag of 2 years (*r*_*S*_ = 0.38 *p* < 0.05) (Figure 5).

#### 3.3.2. UV

We did not find a significant correlation between local received UV (1965–1999) and MS incidence of the region (1970–1999) at the lags of 0 to 5 years (Figure 5).

### 3.4. Nordland County

**Figure 6:**
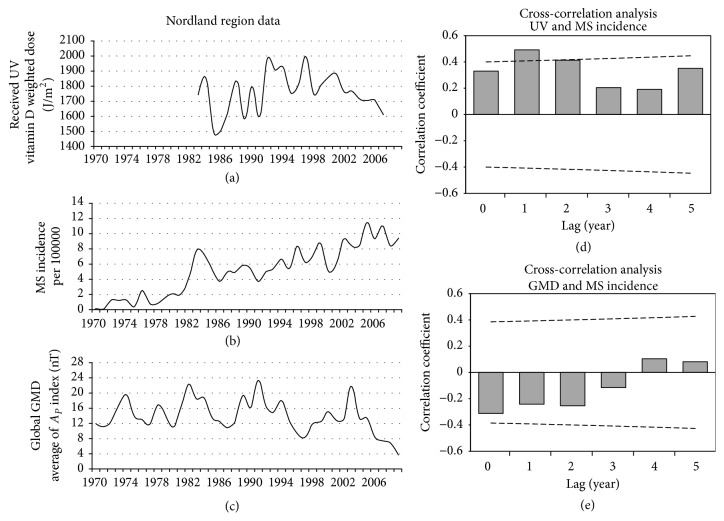
Nordland region data. Panel (a): annual average of the received UV (1983–2007). Panel (b): MS incidence of the Nordland region (1970–2009), adapted from the report of Benjaminsen et al. [15]. Panel (c): annual average of global GMD based on the *A*_*p*_ index (1970–2009). Panel (d): the result of lagged correlation between received UV and MS incidence. Panel (e): the result of lagged correlation between GMD and MS incidence. The dashed lines in those panels (d and e) are the threshold for significant correlation. The horizontal solid lines in these panels indicate coefficient of correlation (*r*) = 0.

No significant correlation was found between Ap and estimated MS incidence (1970–2009) at lags of 0 to 5 years. In contrast, we found a significant positive correlation between received UV (1983–2007) and estimated MS incidence of Nordland (1983–2007) at a lag of 1 year (*r*_*S*_ = 0.49 *p* < 0.05) (Figure 6).

### 3.5. Denmark

#### 3.5.1. GMD

**Figure 7:**
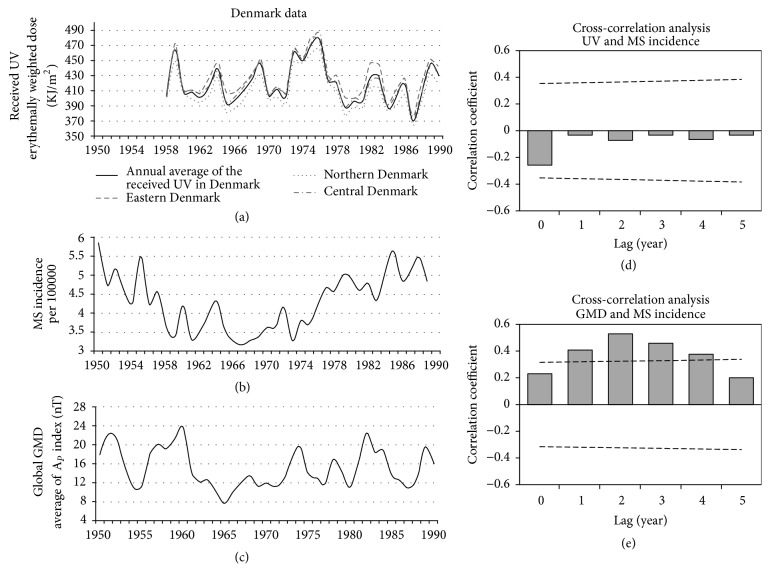
Denmark data. Panel (a): the black line shows the annual average of the received UV (1958–89) calculated from the data of the three sites in the central, northern, and eastern parts of the country (bright gray lines). Panel (b): MS incidence of the Denmark (1950–89), adapted from the report of Koch-Henriksen [16]. Panel (c): annual average of the global GMD based on the *A*_*p*_ index (1950–89). Panel (d): the result of the lagged correlation between received UV and MS incidence. Panel (e): the result of the lagged correlation between GMD and MS incidence. The dashed lines in panels (d and e) are the threshold for significant correlation. The horizontal solid lines in these panels indicate coefficient of correlation (*r*) = 0.

The result of our cross-correlational analysis showed significant positive correlation between Ap and MS incidence in Denmark (1950–1989) at the lags of 1–4 years with maximum positive correlation at the lag of 2 years (*r*_*S*_ = 0.53, *p* < 0.05) (Figure 7).

#### 3.5.2. UV

As mentioned in the methods section, we calculated the received annual UV in the Denmark by averaging the received UV in three locations in northern, eastern, and central part of the country during 1958–1989. We did not find a significant correlation between received UV and MS incidence at lags of 0 to 5 years (Figure 7).

### 3.6. The UK

#### 3.6.1. GMD

**Figure 8:**
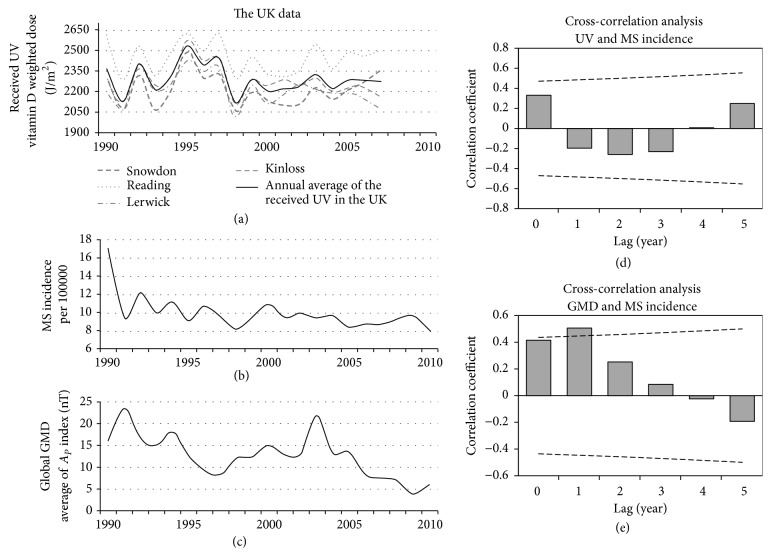
The UK data. Panel (a): the black line shows the annual average of the received UV (1990–2007) calculated from the data of the four sites throughout the UK (bright gray lines). Panel (b): MS incidence of the UK (1990–2010), adapted from the report of Mackenzie et al. [17]. Panel (c): annual average of global GMD based on the *A*_*p*_ index (1990–2010). Panel (d): the result of lagged correlation between received UV and MS incidence. Panel (e): the result of lagged correlation between GMD and MS incidence. The dashed lines in those panels (d and e) are the threshold for significant correlation. The horizontal solid lines in these panels indicate coefficient of correlation (*r*) = 0.

Significant positive correlation was found between Ap index and MS incidence of the UK (1990–2010) with a lag of 1 year (*r*_*S*_ = 0.50, *p* < 0.05) (Figure 8).

#### 3.6.2. UV

Annual received UV throughout the UK was calculated by averaging the received UV data of Snowdown, Reading, Lerwick, and Kinloss from PROMOTE data source. No significant correlation was found between received UV and MS incidence of the UK (1990–2010) (Figure 8).

## 4. Discussion

The GMD and the received UV both are solar-terrestrial related phenomena. During the solar 11-year cycle, both magnetic activity and luminosity of the sun change [26]. The variations of the solar magnetic activity and solar wind are very significant from solar minimum to maximum during a cycle. Coronal mass ejections that rise mainly from active solar regions such as sunspots do not alter overall sun luminosity persistently, but the huge amount of magnetic particles that are released by them can induce significant GMDs or notable geomagnetic storms for hours or days on the Earth. Accordingly, the frequency and severity of GMDs significantly change [2]. In contrast, alterations in solar UV radiation through a cycle are very mild and less than 2% [27].

On the other hand, the effect of Earth on experiencing GMDs and received UV is also notable. The amount of received UV is closely affected by the thickness of atmosphere, climate, cloud thickness, seasons, and the angular distance from the equator. Therefore, in a simple view, by moving from the equator toward the poles (toward higher geographic latitude), the received UV decreases continuously.

In contrast, the amount of experienced GMD is depended mainly on the location on geomagnetic latitude and the space-weather. During the mild to moderate geomagnetic activities, high-latitude areas experience the geomagnetic substorms. The more close to the auroral oval, the more experience of GMDs. On the other hand, during geomagnetic storms, the entire globe will experience notable GMDs, even in the geomagnetic equator [28]. This study found significant positive correlations between alterations in GMDs (Ap index) with the long-term MS incidence in three out of six studied locations. This result is in agreement with the previous study on two locations in the middle latitude areas in Greece and Iran [19] and is in line with the key concept of GMD hypothesis and its basic core that was mentioned in the introduction.

According to the result, the lags between the changes in GMDs and alterations in MS incidences vary mainly from 1 to 2 years. The longer lags are seen in areas that involve older data. We have discussed previously that these lags are not necessarily representative of the exact time lag between the trigger and the consequence [19]. Because of the special nature of the MS, many neurological lesions due to this disease activity may occur in clinically silent areas of the brain. Therefore, there may be a notable delay between the real initiation of the disease and presentation of the first clinical manifestation to cause diagnostic evaluation, confirming the diagnosis and recording the case as a new incidence of the disease. The variations of MS diagnostic criteria also exert important effect. Grytten et al. recently reviewed the effect of the improvement of diagnostic criteria and advent of MRI on the time trends of MS incidence for 50 years in Norway and found that the time from the disease onset to diagnosis confirmation has decreased from a mean time of 10 years to less than 1 year by 2003 [29]. A similar pattern can be seen in our result as there are changes in the lags between GMDs and alterations in MS incidence. We regard it as an indirect clue that the observed lags are not findings just by chance.

We did not find a significant correlation between GMD and MS incidence in the Orkney Islands, Shetland Islands, and Nordland. This issue may be related to the specifications of the MS registries in these locations that have low population or indicate a multifactorial mechanism and the fact that GMDs cannot be the only environmental factor that may play a role in triggering this disease.

Nordland was the only location that showed a significant correlation between received UV and MS incidence. Nevertheless, this correlation is “positive” and means that higher received UV is followed by increase in MS incidence. This finding is in contrast with the concept of VDH.

## 5. Supporting Evidences

Because of the fact that GMD hypothesis for MS is a newborn concept, rare works have been done in this field. Recently, an interesting work has been done by Papathanasopoulos et al. on the relationship between GMDs and patient admissions due to MS attacks [30]. They studied the area that we previously reported its MS incidence association with GMD [11]. Their result confirms such relationship in the level of individual MS attacks. They revealed a more exact time lag between MS and GMDs. Their result pointed out that, shortly after a significant GMD, the MS patient admissions increase significantly [30].

In Canada, in an area near to GML60, Janzen et al. have tested the effect of time of birth on MS risk based on the GMD hypothesis and showed that birth in the years with higher GMD is associated with higher risk of MS in the adulthood [31]. They also found that exposure to GMD during the first decade of life has a cumulative effect on the risk of obtaining MS in adulthood. A similar result has been observed recently by Samoylova et al. in Russia [32].

Another indirect support for GMD hypothesis comes from a recent study by Wing et al. Their subject of study was the possible relationships between solar and geomagnetic indices and rheumatologic inflammatory diseases. They analyzed five decades data of rheumatoid arthritis (RA) and found a significant relationship between the incidence of RA with GMD [33]. Wing et al. not only showed the association between GMD and RA but also discussed how RA prevalence distribution is related to geomagnetic latitude. Genome-wide association studies indicate that RA and MS have shared genetic factors [34]. Both diseases have relapsing-remitting nature, and the effects of environmental factors are evident in their occurrence. Accordingly, we regard the study of Wing et al. as indirect evidence that supports our GMD hypothesis of MS.

## 6. Limitations

In spite of our vast research to find the best long-term UV data for each studied locations in the studied time periods, the coverage of the UV data that we found is shorter than the coverage of the GMD data in this study. Albeit, it should be regarded that the UV data are more exact because those are the results of measurements in the interested regions, but the GMD data is a global index of GMD activity, and the exact GMD alterations in the studied locations were not available. These issues should be considered at the time of comparison of the result between these two hypotheses.

The other limitation of this study is the issue of ecological inference fallacy. The observed correlation in an ecological study does not mean necessarily that such a relationship exists at the individual level. To resolve this issue, studies with higher resolution of MS incidence data are needed. At present, as mentioned before, at least in one of the previously studied locations, such correlation is confirmed at the individual level [11, 30].

Finally, the existence of a significant correlation, even at the individual patient level, does not guarantee the existence of a causal relationship. The underlying mechanism of the effects of GMD on CNS and the immune system is not clear; however, we have discussed elsewhere the most probable mechanisms [18]. Further studies are needed in this regard to elucidate the probable underlying mechanisms of such relationship.

## 7. Conclusion

To the best of our knowledge, it is the first study that compared the possible correlation of solar-terrestrial factors (received UV versus GMD) with long-term alterations of MS incidences in various locations. This study supports the GMD hypothesis and shows that it can describe the long-term alterations of MS incidence more efficiently than the current hypothesis of UV and vitamin D. It does not necessarily mean that we should neglect the discovered effects of vitamin D on the immune system but indicate that there is another environmental factor that potentially may play more important role in triggering this disease. One or two decades ago, we did not know the immune modulatory role of vitamin D. Owing to the vast studies and tries following to the proposal of VDH, many of these aspects were discovered. Now, the result of this study and the hypothesis behind it deserve to be considered as the topic for further research, not only in the field of MS but also in the field of environmental factor research of other similar immune-mediated diseases.
